# Two different short fiber-reinforced resin composites for extensive MOD cavities in premolars and molars

**DOI:** 10.1007/s00784-025-06562-4

**Published:** 2025-09-17

**Authors:** Viivi Oksanen, Jasmina Bijelic-Donova, Pekka K. Vallittu, Lippo Lassila, Sufyan Garoushi

**Affiliations:** 1 Department of Biomaterials Science and Turku Clinical Biomaterial Center –TCBC, Institute of Dentistry, University of Turku, Turku, Finland; 2 Department of Prosthetic Dentistry and Stomatognathic Physiology Institute of Dentistry, University of Turku, Turku, Finland; 3 Wellbeing Services County of South-West Finland, Turku, Finland

**Keywords:** Fracture resistance, Short fiber composite, Bilayered restoration, Extensive restoration, Direct MOD, Tooth size

## Abstract

**Objectives:**

To assess the influence of (1) incorporating micro- and millimeter-scale short fiber-reinforced composite (SFC) on the fracture behavior of large direct restorations made in premolars (P) and molars (M); (2) tooth size on the fracture resistance and their correlation.

**Materials and methods:**

Wide MOD cavities with missing lingual walls were prepared in premolars and molars. Four groups were designed (*n* = 15/group). Teeth in PFC group were restored with particulate filler resin composite (Gaenial Universal Injectable) without fiber reinforcement. Teeth in bilayered group were made of micrometer-scale SFC-core (everX Flow) veneered with a resin composite layer 1 mm in thickness. Monolithic SFC restorations were made either with plain SFC (everX Flow) or with plain hybrid SFC. The hybrid SFC was composed of handmade mixture of micro- and millimeter-scale SFCs (everX Flow and everX Posterior). Specimens were stored in water for 12 months before quasi-static loading. Fractures were analyzed using scanning electron microscopy. Data were statistically analyzed with two-way ANOVA (*p* = 0.05), followed by the Tukey HSD test. Additionally, Pearson correlation analyses (*p* = 0.001) were conducted.

**Results:**

Restorations in premolars exhibited statistically significant lower fracture resistance than those in molars, except for the plain SFC group (*p* < 0.05;*r* = 0.0114) (PFC P 481 ± 159 N; PFC M 1221 ± 435 N; Bilayered P 727 ± 387 N; Bilayerd M 1954 ± 517 N; SFC P 1735 ± 533 N; SFC M 1847 ± 551 N; SFC-hybrid P 1485 ± 339 N; SFC-hybrid M 2280 ± 375 N). In both, premolars and molars, the application of SFC as core or plain restorative material demonstrated superior fracture endurance compared to PFC restorations. Hybrid SFC in molars displayed significantly higher fracture resistance (*p* < 0.05) compared to the other groups. Pearson correlation indicated statistically significant positive correlation in favor of molars (*p* < 0.001;*r* = 0.490) (PFC *r* = 0.712, Bilayered *r* = 0.815, SFC-hybrid *r* = 0.767), except for the plain SFC group (*r* = 0.114).

**Conclusions:**

The volume and consistency of SFC significantly impact the fracture endurance and fracture mode of direct composite restorations.

**Clinical significance:**

Tooth and short fiber reinforced composite (SFC) type are factors that should be taken into account when planning the SFC restoration. Molars could be restored with any type of SFC, however gaining a high volume of plain flowable or preferably hybrid SFC in premolars is highly important.

## Introduction

Several treatment options are available for restoring posterior teeth with moderate to substantial tooth substance loss. Direct and indirect composite restorations, ceramic inlays, onlays and overlays are all considered as very successful modalities to increase tooth longevity, with the risk of failure being higher for non-vital teeth and multi-surface restoration [1]. Building up a non-vital teeth with resin composite substrates and restoring them with ceramic onlays or overlays has been proposed as an alternative option to minimize complications associated with ceramic restorations [1, 2]. Whilst some studies are focused on evaluating the clinical effectiveness of resin composite restorations, whether constructed using direct manual build-up or CAD/CAM techniques [3, 4], other studies directly compare the survival outcomes of direct versus indirect (composite and/or glass-ceramic) restorations [5, 6]. A recent retrospective study with a long-term follow-up and one meta-analysis study both revealed no significant difference in the survival rates of direct and indirect composite cusp-replacing restorations [5, 6]. Similar observation was made in another study evaluating retrospectively a small population treated either with direct bilayered restorations with millimeter-scale short fiber-composite or glass-ceramic endocrowns [7]. In these studies, both approaches were regarded as viable treatment options. However, due to the increased treatment time and higher costs associated with indirect restorations, the direct method may be preferred in many cases [5].

Extensive direct restorations are a complex type of restoration that could be very successful in the hands of an experienced, trained and skillful practitioner [8–10]. The risk for failure of extensive restoration is related to their location in the dental arch. The leading reasons for failure of direct composite restorations in posterior teeth are bulk fractures and wear, which contribute to around 70% of replacements [11]. Acknowledging the complexity of extensive restorations, findings like this raises the question as to whether conventional resin composite should be used alone in high-stress areas where cavities are large and tooth substance is significantly lost. One promising approach to address these challenges is the use of short fiber-reinforced resin composite (SFC), which combines either micro- or millimeter-scale short glass fibers and particulate fillers. SFCs have been designed to improve the resistance to crack propagation and have been a topic of investigation in several laboratory studies [12–15]. SFCs are used as bilayered restorations, in which the SFC material is used as a reinforcement substrate beneath the surface of conventional resin composite [16, 17]. Laboratory investigations have shown that the bilayered structure improves the fracture endurance and preserves the tooth upon fracture [16–20]. These studies indicate that SFC enhances the durability of both, the remaining tooth structure and the composite restoration by serving as a foundation that prevents crack propagation even within the tooth structure itself [19, 20]. However, in order to achieve this effect, the SFC base should be shaped anatomically following the contours of the dentin. Shaped in this way, also called cusp supporting design, it provides support for the cusp and decreases the distance between the SFC and the stress initiation side [19, 21]. This has been shown also in a clinical study. Although conducted on a small population, the study of Bijelic-Donova et al. showed that an anatomically shaped millimeter-scale short fiber-composite base was an effective way to avoid bulk fractures in direct composite restorations [7].

As the reinforcing effect of SFC increases with a higher volume of the material, some researchers have even extended its application to full single structured restorations, where the entire missing structure is reconstructed with SFC only [22]. Until the present time, most studies have assessed the load-bearing capacity of either premolars or molars, and when SFCs are employed, the effect of either the packable (millimeter-scale) or flowable (micrometer-scale) version is assessed. However, comparing both teeth types (premolars and molars) and both SFC-types (micro- and millimeter) and particularly their mixture (hybrid SFC) in the same study set up has yet to be done. Therefore, this study aims to evaluate the impact of incorporating both micro- and millimeter-scale short-fiber composites on the fracture behavior of large direct restorations made in premolars and molars. Furthermore, the study investigates the effect of tooth type on the fracture endurance and the failure mode of these restorations.

## Materials and methods

**Fig. 1:**
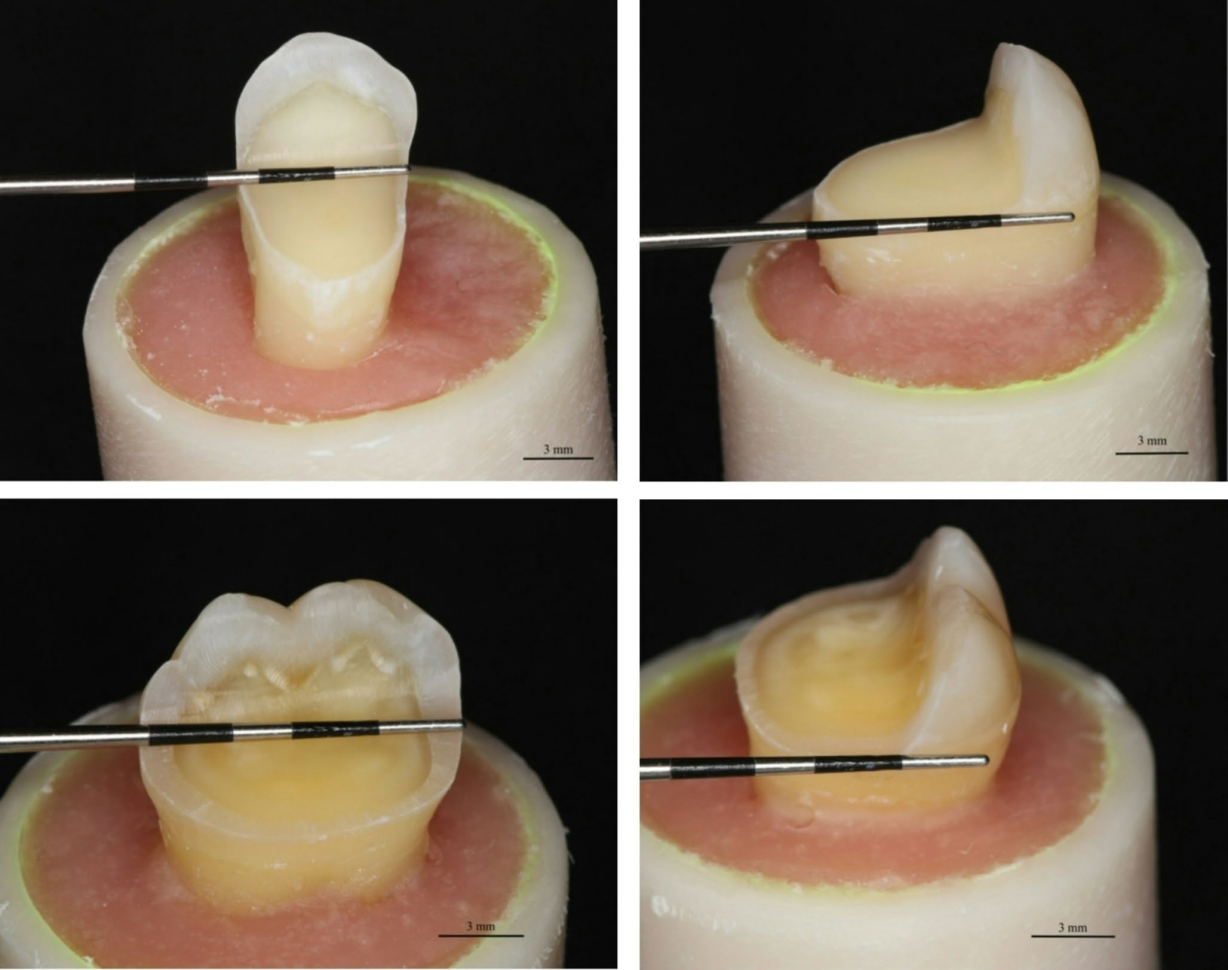
Preparation on premolars and molars showing cavity dimensions in millimeters and the thickness of the remaining buccal wall at its base. **A** and **B**: premolar dimensions: width ≈ 7 mm, length ≈ 11 mm and buccal wall thickness ≈ 3 mm. **C** and **D**: molar dimensions: width ≈ 11 mm, length ≈ 9 mm and buccal wall thickness ≈ 3 mm

The materials used in this study and their composition are listed in Table 1. Sixty (60) sound premolar and sixty (60) sound molar teeth with similar sizes were used. Teeth were extracted from young individuals aged between 12 and 20 years, either due to orthodontic reasons (premolars) or lack of space in the dental arch (molars). The selected teeth were kept in 0.5% chloramine T solution for one month following the elimination of soft tissues under water. A digital caliper (Mitutoyo Corp., Tokyo, Japan) was used to measure each tooth’s size from two sides (bucco-oral and mesio-distal), with size differences averaging only ± 1 mm. Selected and cleaned teeth were placed in one large container, from which they were randomly allocated to different boxes marked with numbers (symbolizing the group number from 1 to 4) and letters (symbolizing the tooth type, P: premolar and M: molar) as follows 1P, 2P, 3P, 4P and 1M, 2M, 3M, 4M. The random allocation was made by picking randomly a tooth from the big container and transferring it to the designed box in consecutive order. Thereafter, teeth were embedded in an acrylic block (diameter 2.5 cm) with an auto-polymerized acrylic resin extending 1 mm beneath the cement-enamel junction (CEJ). Every tooth received a standardized coronal preparation (Fig. 1). The teeth preparation and restorations were performed by a single operator.

**Table 1:** Materials used in the study

Material (code)	Manufacturer	LOT Numbers	Composition
G-aenial Universal Injectable (PFC)	GC Corp, Tokyo, Japan	2311061	Bis-EMA, dimethacrylate co-monomers, UDMA, silica, barium glass, (filler content: 69 wt%/45 vol%)
everX Flow, Bulk shade (micrometer-scale, flowable SFC)	GC Corp, Tokyo, Japan	2311281	Bis-EMA, TEGDMA, UDMA, micrometer scale glass fiber filler, barium glass (filler content: 70 wt%, 46 vol%). The Bulk shade displays a depth of cure of 5.5 mm
everX Posterior (millimeter-scale, packable SFC)	GC Corp, Tokyo, Japan	2310241	Bis-GMA, PMMA, TEGDMA, millimeter scale glass-fiber filler, barium glass (filler content: 76 wt%, 57 vol%)

*PFC *particulate filler composite, *SFC* short fiber composite, *TEGDMA* triethylene glycol dimethacrylate, *UDMA* urethane dimethacrylate, *Bis-GMA* bisphenol A glycol dimethacrylate, *PMMA* poly(methyl) methacrylate, *Bis-EMA* Ethoxylated bisphenol-A-dimethacrylate, wt%: weight percentage, vol% percentage by volume

### Tooth preparation and restoration methods

The preparation and restoration procedures used in this study followed a previously described approach [23, 24], and the sample size of 15 teeth per group was selected based on the literature, without a formal sample size calculation. Standardized tooth preparations were performed to replicate large mesio-occluso-distal (MOD) cavities with the lingual cusps removed. Measurements were taken using a periodontal probe and standard burs to ensure precise dimensions. The preparations were completed using a high-speed handpiece with round-end diamond burs (850 − 014 M SSWhite, Lakewood, NJ, USA) under water cooling. The cavity floor was flat, with an occlusal reduction of 5 mm and an average remaining buccal wall thickness of approximately 3 mm (Fig. 1). The restoration margins were placed around 1–1.5 mm above the CEJ. Teeth were restored following the same adhesive protocol. During the adhesive process, the enamel was selectively etched with 37% phosphoric acid (Scotchbond Universal etchant, 3 M ESPE, Seefeld, Germany) for 15 s and then rinsed. After air-drying, a one-step universal adhesive system (G-Premio Bond, GC Europe, Leuven, Belgium) was applied using a microbrush-X disposable applicator (Pentron Clinical Technologies, LLC, USA), then air-thinned and light cured for 20 s with an Elipar TM S10 light- curing unit (3 M ESPE). The light-curing unit produced irradiance of an average 1600 mW/cm² and a wavelength range of 430–480 nm. The curing light tip was held in close proximity to the surface. Teeth (*n* = 15 premolars and *n* = 15 molars per material group) were then restored according to the group to which they belong. Prior to preparation, a translucent mold was made (Exaclear, GC Europe) from each tooth to aid standardized restoration fabrication, and a stamp to restore the original occlusal anatomy.

Premolar (P) and molar (M) teeth in the control PFC group (PFC: particulate filler composite; PFC P and PFC M), were restored using conventional resin composite (G-aenial Universal Injectable, GC Corp., Tokyo, Japan) without any fiber reinforcement. The resin composite was applied using a horizontal layering technique, with each layer being approximately 2 mm thick. The occlusal layer of resin composite was placed with the stamp to restore the original occlusal anatomy. Each increment was light-polymerized for 40 s. Teeth in the bilayered group (Bilayered P for premolars and Bilayered M for molars) were restored using flowable, micrometer-scale SFC (everX Flow, Bulk Shade, GC Corp.) as core material (4 mm) veneered with a 1 mm layer of conventional resin composite (G-aenial Universal Injectable, GC Corp., Tokyo, Japan). The SFC-core was applied in bulk increment, but shaped anatomically and light-polymerized for 40 s. The SFC-core thickness was ensured with a scaled periodontal probe and guided by the translucent mold cut in half to serve as an index. The occlusal layer of resin composite was placed with the stamp as mentioned previously. Premolar (P) and molar (M) teeth in the plain SFC group (SFC P, SFC M) were restored using plain micrometer-scale SFC (everX Flow, Bulk Shade, GC Corp., Tokyo, Japan) without any surface coverage. The material was applied and light-polymerized in bulk (40 s) using the translucent mold and 40 s per side upon removing the mold. Premolar (P) and molar (M) teeth in the plain SFC-hybrid (SFC-hybrid P, SFC-hybrid M) were restored using both micro- and millimeter-scale SFCs (everX Flow, Bulk Shade and everX Posterior, GC Corp., Tokyo, Japan) without any surface coverage. Both materials were hand mixed and applied as a mixture in increments of 2 mm each with the aid of the translucent mold cut in half to serve as an index. The occlusal anatomy was created with the stamp pressed over the final layer. Each layer was light-polymerized for 40 s. Upon finishing and polishing, restored teeth were stored in distilled water at 37 °C for 12 months to simulate the aging process that occurs under oral conditions before testing.

### Fracture load test

Restorations were submitted to a quasi-static load at room temperature using a universal testing machine (Lloyd model LRX, Lloyd Instruments Ltd, Fareham, UK) at a rate of 1 mm/min. A 5 mm diameter metal ball was used to apply the load vertically between the triangular ridges of the lingual and buccal cusps. The loading process was carefully observed until restoration fracture occurred, as indicated by the final incline in the load-deflection curve. This process was done by experienced operator unaware of the group being tested. Subsequently, three researchers visually examined the fracture modes of each loaded restoration, classifying them into two types: **repairable** cohesive fractures limited to the restoration and/or tooth, above the CEJ, such as chipping or delamination within the restorative material; and **catastrophic (non-repairable)** fractures with fracture line extending to the root or below the CEJ, making the tooth non-restorable.

### Fracture mode and microstructure analysis

The restorations’ fracture mode was assessed visually and with stereomicroscopy at different magnifications and illumination angles (Heerbrugg M3Z, Heerbrugg, Switzerland). After examining the specimens independently, three researchers came to an agreement regarding the type, location, and direction of failure. Representative fractured specimens were inspected using SEM (LEO, Oberkochen, Germany) for fractographic evaluation. Each specimen was gold coated prior to observation, using a sputter coater (BAL-TEC SCD 050 Sputter Coater, Balzers, Liechtenstein) and a vacuum evaporator.

### Statistical analysis

The data were analyzed using SPSS version 23 (Statistical Package for Social Science, SPSS Inc, Chicago, IL, USA) using analysis of variance (ANOVA) (*p* < 0.05) followed by a Tukey’s *post hoc* method. Additional Pearson correlation was used to evaluate the correlation between the tooth type and the fracture load as well as between the material type, the tooth type and the fracture load.

## Results

**Fig. 2:**
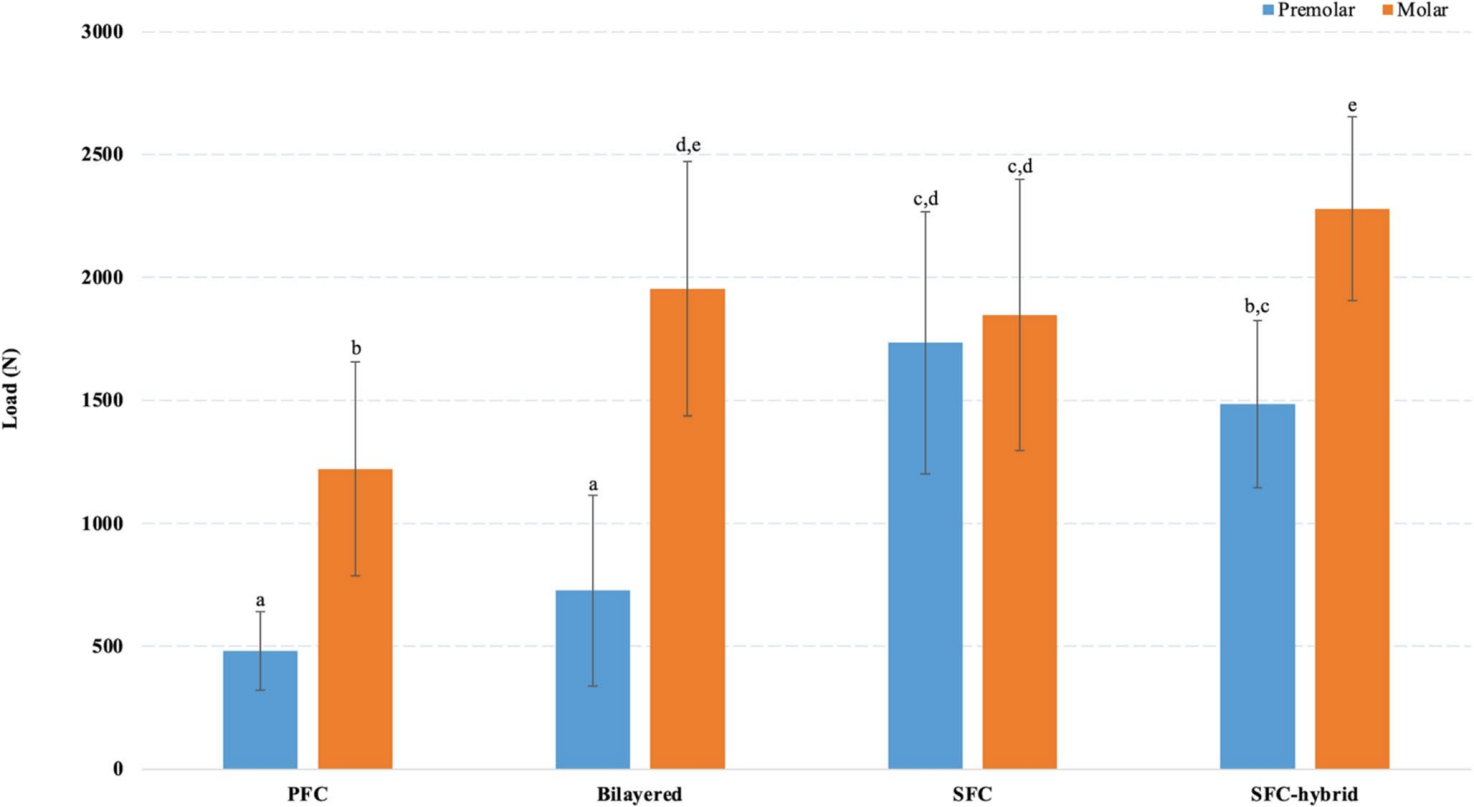
Mean final fracture load (*N*) and standard deviation (*SD*) of tested restorations. Different letters indicate significant differences (*p* < 0.05). **PFC**: particulate filler composite; **SFC**: short fiber composite

The restorative technique significantly impacted the fracture resistance of the assessed restorations (*p* < 0.05). Levene’s test confirmed that variances were equal and homogeneous across the groups. Figure 2 presents the average fracture load values with standard deviations for the tested restorations (PFC P 481 ± 159 N; PFC M 1221 ± 435 N; Bilayered P 727 ± 387 N; Bilayerd M 1954 ± 517 N; SFC P 1735 ± 533 N; SFC M 1847 ± 551 N; SFC-hybrid P 1485 ± 339 N; SFC-hybrid M 2280 ± 375 N). In general, the use of any type of SFC, either as a core material or as a standalone restorative material, showed improved fracture resistance compared to non-fiber reinforced restorations (control PFC group). Premolar restorations exhibited statistically lower fracture resistance values (*p* < 0.05) compared to molars, except for the plain SFC group. ANOVA analysis indicated that the difference between premolars and molars restored with plain micrometer scale SFC (everX Flow, Bulk Shade) was not statistically different (*p* > 0.05). Moreover, bilayered molar restorations (Bilayered M) were also statistically similar to those made with either plain SFC (SFC M) or SFC-hybrid (SFC-hybrid M) (*p* > 0.05), but this was not the case for premolars, which differed significantly among each other (*p* < 0.05) (Fig. 2). In premolars, the effect of bilayered restoration was insignificant, whereas the effect of plain micrometer- or hybrid SFC was statistically strong (*p* < 0.05). However, molar hybrid restorations utilizing a mixture of micro- and millimeter-scale SFCs (SFC-hybrid M) demonstrated significantly higher fracture resistance values (2280 ± 375 N) (*p* < 0.05) compared to any other tested groups.

Expressed otherwise, in molars, the difference between the SFC groups (bilayered M, SFC M, SFC-hybrid M) and control material (PFC M) was statistically significant, but the difference between the SFC reinforcement types was not. However, in premolars, only the difference between the plain SFC groups (SFC P, SFC-hybrid P) and control material (PFC P) was statistically significant, but not between the bilayered SFC (bilayered P) and the control (PFC P) group. Hence, the evidence that bilayered restorations in premolars are statistically better than resin composite is weak (*p* > 0.05).

Pearson correlation analysis was computed to evaluate the association between the tooth type and the fracture load. According to these results, molars generally exhibit higher fracture load values than premolars (*r* = 0.490; *p* < 0.001) which indicates a moderate, statistically significant positive correlation in favor of molars. However, if tooth- and material groups are analyzed individually, it appears that molars show stronger evidence that any type of SFC is superior over resin composite, and that hybrid SFC substrate provides best fracture resistance (2280 N; *r* = 0.767). Observed Pearson correlation values were *r* = 0.712 (PFC), *r* = 0.815 (Bilayered) and *r* = 0.767 (SFC-hybrid). However, the Pearson correlation for the plain SFC group was *r* = 0.114, which indicates statistically non-significant difference between premolars and molars.

**Fig. 3:**
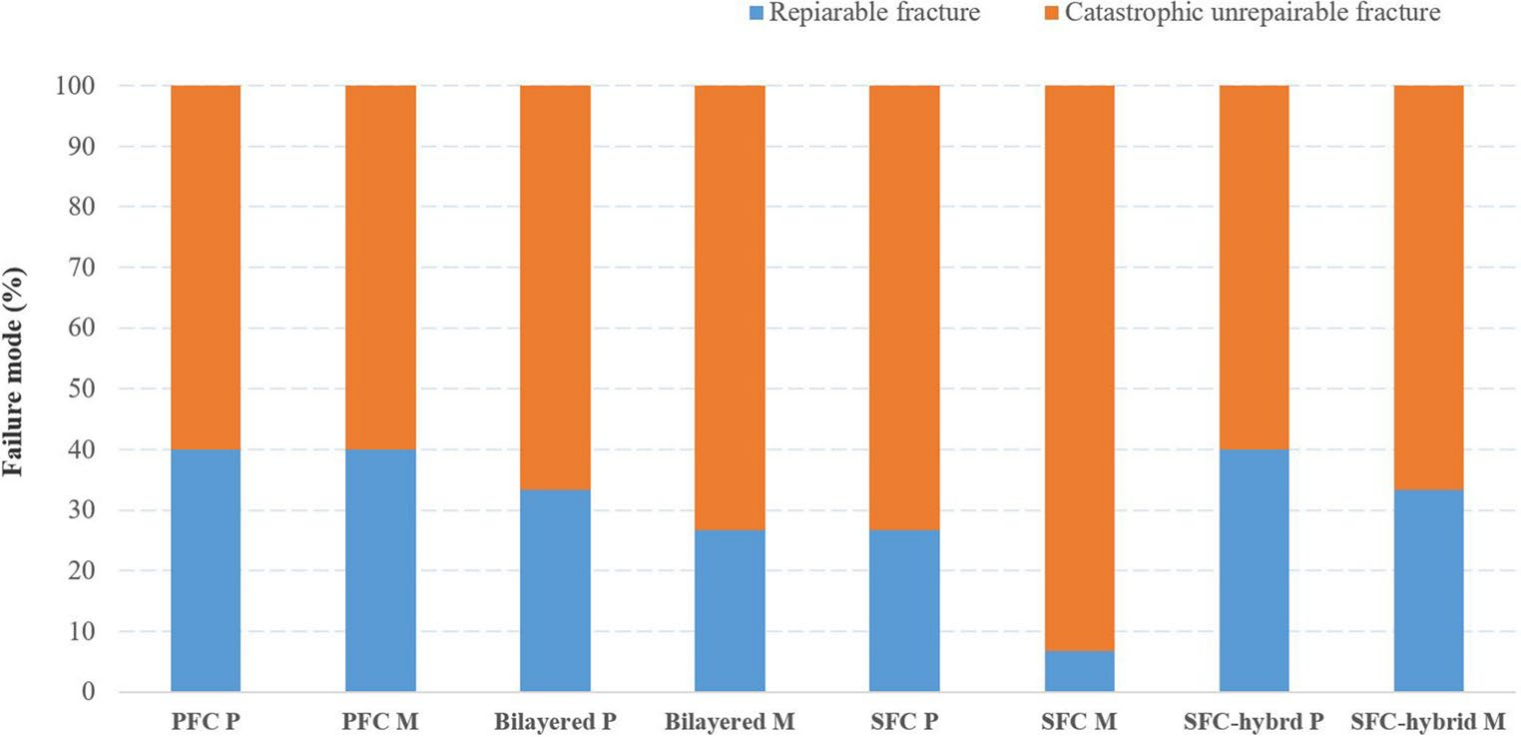
Fracture type analysis presented as diagram with percentage values for the reparable and catastrophic fractures for each tooth and material type. **PFC**: particulate filler composite; **SFC**: short fiber composite; **P**: premolar,** M**: molar

**Fig. 4:**
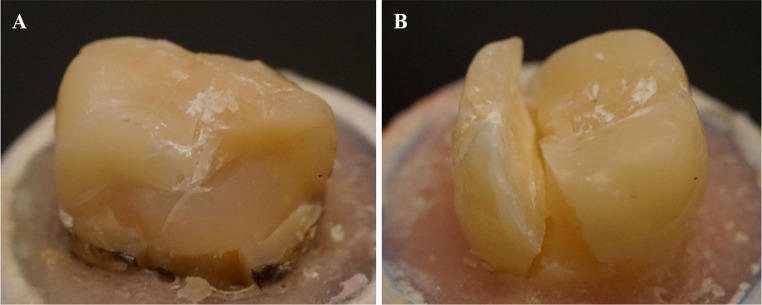
Images of the typical repairable (**A**) and catastrophic unrepairable (**B**) fractures modes of tested restorations

Figure 3 shows the results from the fracture type analysis. Most of the restorations, regardless of the restorative material, fractured catastrophically (60–90%), but this failure type was principally representative for molar restorations restored with plain SFC (everX Flow, Bulk Shade). Figure 4 shows images of the typical fractures.

**Fig. 5:**
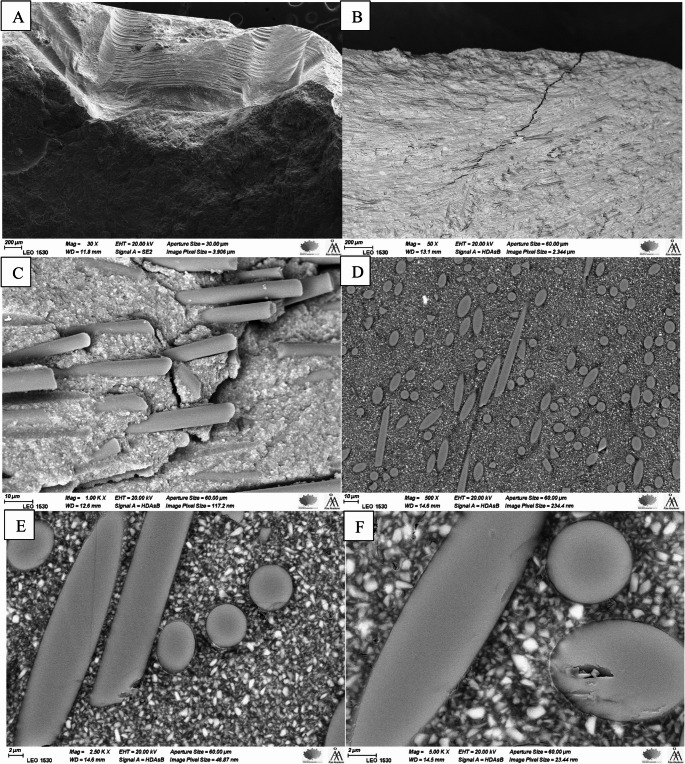
SEM images of the fracture surfaces of the investigated SFC restorations observed at different magnifications (**A**-**C**). **A**: illustrates crack initiation place. **B**: illustrates the short fibers’ capability to redirect crack propagation; **C**: illustrates protruding fibers at the fracture surface *i.e.* fiber pull out. Images (**D**–**F**) show the microstructure of the SFC-hybrid restoration, where both micro- and millimeter-scale short fibers were incorporated. SEM analysis revealed that the micrometer-scale fibers filled the spaces between the millimeter-scale fibers, with both types oriented at varying angles

Figure 5 displays SEM images (A-C) of representative fractures in SFC specimens. These images, taken at different magnifications, demonstrate fiber pull-out from the fracture surface and redirection of crack propagation by the short fibers. Images D-F show the microstructure of the SFC-hybrid restoration, where both micro- and millimeter-scale SFCs were incorporated.

## Discussion

The present research was designed to evaluate the effect of using a mixture of micro- and millimeter-scale short-fiber composites in restoring directly large MOD restorations in premolars and molars. Teeth restored with the 50/50 mixture of micro- (everX Flow, Bulk Shade) and millimeter-scale (packable everX Posterior) short-fiber composites were named hybrid SFC restorations (SFC-hybrid group). Although the results for premolars were less clear than for molars, the outcome that hybrid SFC improves the fracture resistance of large restorations and their failure mode is strong. Indeed, only the hybrid SFC had the potential to increase the likelihood for restorable fractures. These findings align with previous research demonstrating that the use of discontinuous fiber fillers of varying lengths (*i.e.* hybrid approach) in a polymer matrix improves mechanical performance compared to commercial SFC and conventional posterior resin composites [25]. This improvement may result from a synergistic effect between the two fiber sizes, where micrometer-scale fibers fill the gaps between millimeter-scale fibers, promoting better crack deflection and stress distribution throughout the restoration. However, manual blending of these materials carries the risk of void incorporation, which may act as stress concentrators and compromise fracture endurance [26, 27].

The correlation analysis showed that the fracture resistance was dependent and correlated to the tooth size. Molar teeth withstood higher load values than premolars (*r* = 0.490; *p* < 0.001) and the difference among the SFC types for molars was less obvious than for premolars. In other words, this means that clinically, the tooth type is a factor that should be taken into account when planning and selecting the SFC material and restoration procedure. While molars could be restored with any type of SFC, gaining a high volume of plain or preferably hybrid SFC in premolars is of primary importance.

The similar performance for both tooth types restored with plain micrometer-scale SFC (SFC P and SFC M) suggests that the structural uniformity was easier to achieve with the flowable SFC. This could mean that the structural homogeneity attained by the SFC material is more relevant factor than the size of the tooth it is used to restore or having various SFC material combinations within the restorations. However, this observation has to be taken with caution, because the mixing of the SFCs in this study was done manually. Namely, for the purposes of this study, micro- and millimeter scale fiber composites were hand-mixed and then applied into the cavity. This process could introduce voids. Indeed, the absence of synergetic properties of the SFC mixture due to void entrapment was observed in another study [26]. Magne et al. showed that the 50/50 mixture of micro- and millimeter-scale SFCs (hybrid restorations) showed intermediate performance between the micrometer (everX Flow) and millimeter-scale (everX Posterior) versions. Such a correlation cannot be done in this investigation, because a group with millimeter-scale SFC (everX Posterior) is missing. Consequently, this is a limitation of the present study.

In this investigation, only the hybrid SFC had a potential to increase the likelihood of restorable fractures. Micrometer scale SFC (everX Flow) used with veneering composite or alone (Bilayered and plain SFC groups) did not protect the tooth structure from catastrophic fractures. This finding is in line with the finding in another study, which showed that the micrometer-scale short fibers in EverX Flow have an ability to divert the crack path, but only long-aspect ratio (that is millimeter-scale) short fibers as in everX Posterior could stop the crack from propagating [13]. Contrary to the assumption that a thick layer of micrometer scale short fibers could alleviate these events, it seems that low-aspect ratio fibers have limitations in terms of arresting a crack. Although the fiber pull out mechanism of micro-meter short fibers increases the toughness due to the frictional energy, this mechanisms alone is not enough to impede the crack [13, 28]. Perhaps the thick core of the micrometer scale SFC should be made incrementally and not in bulk layer [17]. The hybrid SFC could overcome this shortcoming most likely due to the co-adjuvant effect of fiber-pull out and fiber breakage mechanisms provided by the micro- and the millimeter-scale short fibers, respectively (Fig. 5). This assumption is supported by the finding of another study, that the high viscosity of the short fiber-reinforced resin composite is crucial for obtaining high resistance to fracture and more favorable failures [17].

The clinical relevance of the mode of fracture is better recognized when SFC groups are compared to conventional resin composite restorations (PFC groups). The hybrid SFC restorations (SFC-hybrid groups) resulted mainly in repairable fractures, whereas the conventional composite restorations (PFC groups) showed mainly catastrophic fractures. This becomes an important finding for the clinician, because clinically, direct restorations are predominantly made from particular filler composite without fiber reinforcement. By improving the chances for repairable fracture, hybrid SFC present an easy and practical method for preventing tooth loss and preserving natural tooth structure. Indeed, repairable fractures extend the functional life of restorations and minimize the need for extensive interventions.

Micrometer-scale SFC (everX Flow, Bulk Shade), whether used with a veneering composite or as a monolithic restoration (Bilayered and plain SFC groups), exhibited lower fracture resistance compared to the hybrid SFC combination. This suggests that while fiber reinforcement contributes to improved fracture resistance, the specific composition and structural characteristics of the SFC—such as fiber length, orientation, and distribution—are critical for achieving optimal performance. Additionally, the presence of PMMA in the hybrid SFC composition (Table 1) may play a role in its enhanced behavior by introducing flexibility or a plasticizing effect to the highly cross-linked resin matrix, potentially improving energy absorption during loading [29]. Future studies should further investigate these variables to optimize the mechanical properties and clinical performance of next-generation SFC materials.

Limitations of this study include the general limitations of in vitro study, such as lacking the temperature fluctuation, moisture and dynamic occlusal forces occurring in the oral cavity. The load-to-fracture test is a simple, but destructive test in nature, and results in higher loads and more destructive failures. The events observed in this type of test could be regarded as worst-case-scenario cases, where failures occur after impact, trauma, parafunctions and bruxism. Despite the shortcomings, the load-to-fracture test could still be utilized to predict the clinical behavior of a material, because it gives insights into its fracture resistance. More specific limitations of this study are the absence of a quantitative topography to examine the features of the fracture surfaces, a control group (intact teeth) and a group with millimeter-scale SFC (everX Posterior). On other hand, in this investigation, premolars and molars with the same design and material combinations were used. This set up could be seen as a strength of this research. Namely, it has been recently reported that while there is strong evidence to support the beneficial effect of fiber-reinforcement in molars, the heterogeneity of studies in premolars is high and contributes to making inconclusive results on the benefit of using fiber-reinforcement [21]. Reducing the methodological heterogeneity in the present research allowed the assessment of the true effect of the short fiber reinforcement, which could be considered a key strength of this in vitro investigation. Future studies are warranted to evaluate the effect of different SFC bases under indirect restorations preferably in a fatigue type of set up, which better replicates the complex intraoral conditions.

## Conclusion

The volume and consistency of short fiber reinforced composite (SFC) used in large cavities significantly impact the fracture endurance and fracture mode of direct composite restorations. When planning a direct SFC restoration, both the type of tooth to be restored and the type of SFC should be taken into account. Molars could be restored with any type of SFC, however gaining a high volume of plain flowable or preferably hybrid SFC in premolars is highly important.

## Data Availability

Data are available from S.G. upon request after approval by all authors.
